# Bias-Voltage Stabilizer for HVHF Amplifiers in VHF Pulse-Echo Measurement Systems

**DOI:** 10.3390/s17102425

**Published:** 2017-10-23

**Authors:** Hojong Choi, Chulwoo Park, Jungsuk Kim, Hayong Jung

**Affiliations:** 1 Department of Medical IT Convergence Engineering, Kumho National Institute of Technology, Gumi 39253, Korea; hojongch@kumoh.ac.kr (H.C.); pcw3108@kumoh.ac.kr (C.P.); 2 Department of Biomedical Engineering, Gachon University, In-cheon 21936, Korea; 3 National Institute of Health Transducer Resource Center and Department of Biomedical Engineering, University of Southern California, Los Angeles, CA 90089, USA

**Keywords:** bias-voltage stabilizer, high-voltage–high-frequency amplifier, VHF ultrasound transducers, pulse-echo measurement systems

## Abstract

The impact of high-voltage–high-frequency (HVHF) amplifiers on echo-signal quality is greater with very-high-frequency (VHF, ≥100 MHz) ultrasound transducers than with low-frequency (LF, ≤15 MHz) ultrasound transducers. Hence, the bias voltage of an HVHF amplifier must be stabilized to ensure stable echo-signal amplitudes. We propose a bias-voltage stabilizer circuit to maintain stable DC voltages over a wide input range, thus reducing the harmonic-distortion components of the echo signals in VHF pulse-echo measurement systems. To confirm the feasibility of the bias-voltage stabilizer, we measured and compared the deviations in the gain of the HVHF amplifier with and without a bias-voltage stabilizer. Between −13 and 26 dBm, the measured gain deviations of a HVHF amplifier with a bias-voltage stabilizer are less than that of an amplifier without a bias-voltage stabilizer. In order to confirm the feasibility of the bias-voltage stabilizer, we compared the pulse-echo responses of the amplifiers, which are typically used for the evaluation of transducers or electronic components used in pulse-echo measurement systems. From the responses, we observed that the amplitudes of the echo signals of a VHF transducer triggered by the HVHF amplifier with a bias-voltage stabilizer were higher than those of the transducer triggered by the HVHF amplifier alone. The second, third, and fourth harmonic-distortion components of the HVHF amplifier with the bias-voltage stabilizer were also lower than those of the HVHF amplifier alone. Hence, the proposed scheme is a promising method for stabilizing the bias voltage of an HVHF amplifier, and improving the echo-signal quality of VHF transducers.

## 1. Introduction

Conventional ultrasound machines in current use in hospitals and laboratories work in the low-frequency (LF) range, between 2 MHz and 15 MHz, and have millimeter-range resolution [1,2]. A primary component of ultrasound systems is the ultrasound transducer, which converts electrical energy to acoustic energy or vice versa [3,4,5]. An amplifier is used to excite the ultrasound transducers to generate acoustic waves for transmission to a target through a medium such as human tissue or blood vessels [2]. The acoustic waves reflected back to the transducers are converted to electrical signals [6,7]. The weak electrical signals are then amplified by a low-noise preamplifier and a time-gain-compensation amplifier [8]. Very-high-frequency (VHF) ultrasound has recently been developed for use in applications such as acoustic microscopy and micro-beam stimulation, owing to the higher spatial resolutions of these techniques in comparison to LF ultrasound applications such as non-destructive testing [9,10,11]. As the operating frequency of a ultrasound transducer increases, the size decreases accordingly [7]. In response to this reduction in size, the range of the maximum output voltage and supply voltage of the high-voltage–high-frequency (HVHF) amplifier used for triggering the transducer is also assumed to decrease [7,12]. Hence, the amplitudes of the echo signals of a VHF ultrasound transducer are affected by the performance of the ultrasound HVHF amplifier [6,13]. In addition, the echo-signal amplitudes of a VHF ultrasound transducer are reduced, in comparison to the amplitudes of a corresponding LF device, because of the limited voltage range of the HVHF amplifier and the cable loading effects [14,15]. The parasitic capacitances and inductances of the metal-oxide-semiconductor field-effect transistor (MOSFET) devices and coaxial cables used in the amplifier can also affect its performance in the high-frequency and high-voltage ranges [16]. Therefore, bias-voltage control is crucial in defining the HVHF amplifier performance.

HVHF amplifiers for use in ultrasound transducers are required to be linear amplifiers with low harmonic distortions, as this characteristic affects the echo-signal quality of the ultrasound transducers [13,17]. In particular, the coded excitation method for ultrasound harmonic imaging requires low harmonic distortions in the amplifier [7]. Push–pull type amplifiers have been used to reduce even-order harmonics [18,19]. This architecture requires the use of transformers at the input and output of the amplifier to combine the differential signals. However, the transformers can increase the ring-down of the echo signals generated by the ultrasound transducers, as the VHF ultrasound transducers are more sensitive to cable loading and inductive effects than LF ultrasound transducers [20]. A bi-polar digital pulser driven by a level shifter has also been used. However, the operating frequency of HVHF amplifiers is limited by the bandwidth of the digital–analog converter in the field programming gate array chip, and the harmonic distortion of the amplifier is affected by the performance of the level shifter [13]. Therefore, single-ended amplifier architectures are preferable for integration with VHF pulse-echo measurement systems.

As an unstable output voltage from the amplifier affects the performance of an ultrasound transducer, the bias voltage of the amplifier must be stabilized [13]. In the low-frequency operation, circuits consisting of the bias inductors, bypass capacitors, or transformers have typically been used for bias-voltage stabilization. These circuits constitute a simple biasing method, operating within a narrow range at low frequencies owing to the variation of inductance at different frequency ranges [18,19,21,22,23]. These circuit topologies are not suitable for high-frequency operation, as the non-linear components from the high-frequency input signal cannot be impeded from the main supply voltage [18,23]. Voltage regulators have also been used to stabilize the bias voltage of an amplifier to minimize signal distortions. However, with this technique, the bias voltages can be affected by the resistance selected for the resistor-divider circuit, because of the variations in resistance induced during HV operation [17].

In this paper, we propose a bias-voltage stabilizer using an HV MOSFET with resistor, capacitor, and inductor, dedicated for VHF ultrasound transducers. Similar techniques such as active voltage feedback schemes and MOSFET bias schemes have been used to bias the voltage of amplifiers [19,24,25]. These bias schemes were originally designed for low-voltage (LV) wireless communication systems, and therefore may not be directly applicable to ultrasound transducer applications that require HV operations.

We expect that the bias-voltage stabilizer circuit can stabilize the wide input power range of HVHF amplifiers. Hence, HVHF amplifiers with bias-voltage stabilizers can reduce unwanted harmonic distortions of the echo signals in pulse-echo measurement systems to improve the echo-signal quality [7]. The remainder of this paper is structured as follows: the design and implementation of an HVHF amplifier with a bias-voltage stabilizer and voltage regulator are described in Section 2; the operating mechanism of the bias-voltage regulator and a comparison of the performance of HVHF amplifiers with and without the bias-voltage stabilizer and voltage regulator are given in Section 3, where we also demonstrate that the bias-voltage stabilizer can reduce harmonic distortion components in pulse-echo measurement systems; and conclusions of the paper are presented in Section 4.

## 2. Materials and Methods

**Figure 1:**
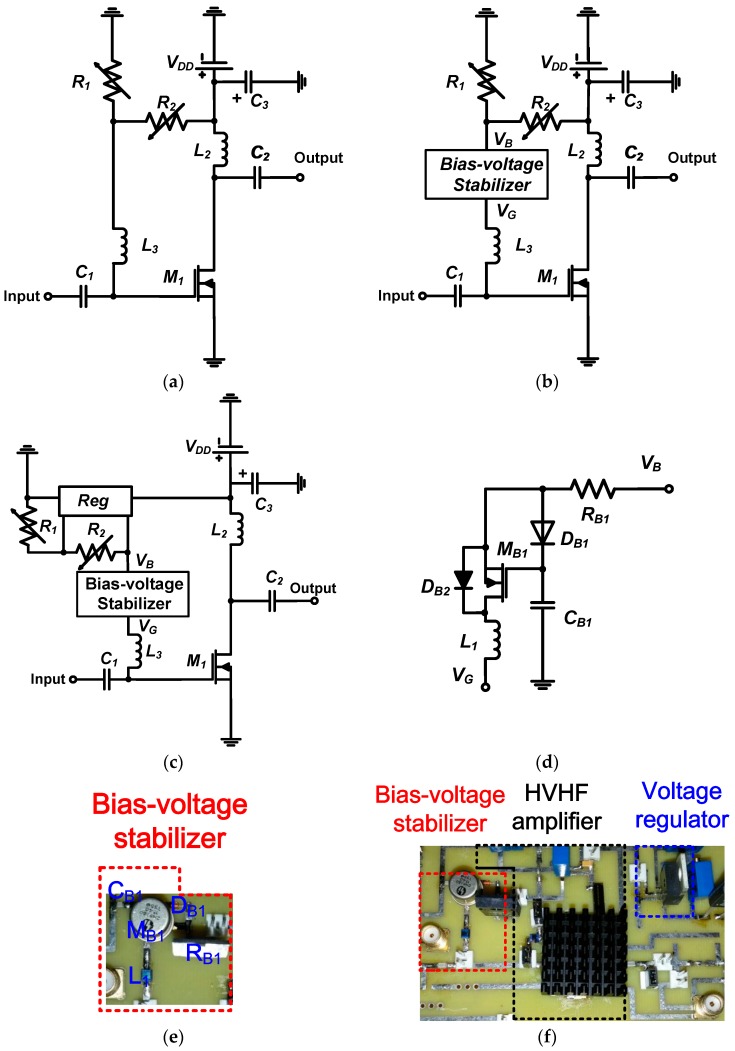
Architectures of (**a**) the high-voltage–high-frequency (HVHF) amplifier; (**b**) the HVHF amplifier with the bias-voltage stabilizer; (**c**) the HVHF amplifier with the bias-voltage stabilizer and a voltage regulator; and (**d**) the bias-voltage stabilizer. Implementation of (**e**) the bias-voltage stabilizer; and (**f**) an HVHF amplifier with the bias-voltage stabilizer and a voltage regulator.

The design of an HVHF amplifier for VHF ultrasound transducers is made challenging by the fact that the operation of MOSFETs in the high-frequency and high-voltage range is poorly defined, and parameters such as gain and bandwidth are difficult to predict using simulation models [15,23]. Therefore, we directly implemented our proposed HVHF amplifier into a multi-layer printed circuit board with an FR-4 insulating substrate and 4-mm copper conducting layers to reduce the parasitic capacitances between the electronic components. Figure 1a,b show the architectures of an HVHF amplifier, without and with a bias-voltage stabilizer, respectively. Figure 1a depicts a typical structure of a simple bias-voltage stabilizer for operating in the low-frequency range. Figure 1c,d show the proposed architectures of an HVHF amplifier with both the bias-voltage stabilizer and a voltage regulator, and of the bias-voltage stabilizer, respectively. The implementations of the proposed bias-voltage stabilizer, and of the HVHF amplifier with a bias-voltage stabilizer and a voltage regulator are shown in Figure 1e,f, respectively. The HVHF amplifier is a typical Class-A- amplifier [19]. A heat-sink was attached to the main HV transistor (M_1_) and voltage regulator (*Reg*) in order to regulate the operating temperature of these devices.

We used a radio frequency (RF) choke inductor, *L*_2_, (Coilcraft, Inc., Silver Lake Road, IL, USA) in the HVHF amplifier (Figure 1a) to minimize the voltage drop and achieve maximum output voltage amplification from the main transistor, *M*_1_ (MRF136, MACOM, Lowell, MA, USA) [19]. Input and output coupling capacitors, *C*_1_ and *C*_2_ (AVX Corp., Fountain Inn, SC, USA), were used to block the voltages generated from the DC power supply. The bias inductor, *L*_3_ (Coilcraft, Inc., Silver Lake Road, IL, USA), was used to block unwanted RF signals from the input. An electrolyte capacitor, *C*_3_, (AVX Corp.) was used to minimize the voltage drop from the power supply (*V*_DD_). The rating of this capacitor was selected to be higher than the power supply voltage. The supply voltage (*V*_DD_) generated by the DC power supply was applied to the variable resistor divider (*R*_1_ and *R*_2_, Vishay Siliconix, Silver Lake Road, IL, USA), as shown in Figure 1b. The voltage regulator in Figure 1c was connected to the bias-voltage stabilizer (*V*_B_). The DC voltage was supplied either from the output of the resistor divider or the voltage regulator (*Reg*, LM2931CT, Texas Instrument, Dallas, TX, USA), as shown in Figure 1c. The voltage regulator in Figure 1c was used to maintain a constant voltage. However, we expected that the regulator alone cannot stabilize the bias voltage completely over the wide input range of the HVHF amplifier. We installed a resistor (*R*_B1_, Caddock Electronics Inc., Riverside, CA, USA), a diode (*D*_B1_, 1N4148, Diodes Inc., Plano, TX, USA), a capacitor (*C*_B1_, AVX Corp.), and an HV MOSFET (*M*_B1_, 2N6660, Vishay Siliconix) in the bias-voltage stabilizer (Figure 1d) to block unwanted bipolar RF input signals, in order to stabilize the DC bias voltage from the input (*V*_B_) of the DC power supply. As unipolar or bipolar signals are typically applied in the ultrasound systems, we used cross-diode combinations (*D*_B1_ and *D*_B2_) in the bias-voltage stabilizer.

**Figure 2:**
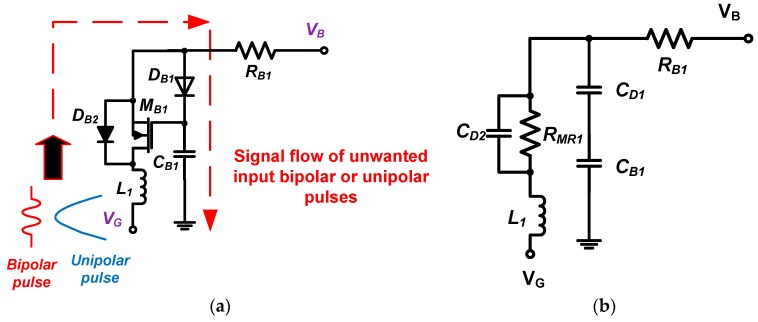
(**a**) Signal flow of unwanted input pulses through the proposed bias-voltage stabilizer; (**b**) Equivalent circuit model for the bias-voltage stabilizer.

To describe the bias-voltage stabilization mechanism, the signal path for unwanted input bipolar or unipolar pulses through the stabilizer is shown in Figure 2. The leakage voltage at point *V*_B_ is expected to be negligible when unwanted input bipolar or unipolar pulses are applied to the HVHF amplifier at the point *V*_G_. Thus, these unwanted pulses are directed to ground. In ultrasound applications, both bipolar and unipolar input signals are applied to the amplifier. Hence, the two diodes (*D*_B1_ and *D*_B2_) are connected in cross-coupled architectures.

Using the equivalent circuit model of the bias stabilizer, we can calculate the values of the components in the circuit when a specific input pulse frequency is required in the HVHF amplifier. The impedances of the inductor (*L*_1_), resistor (*R*_B1_), and capacitor (*C*_B1_) at the operating frequency (*f*_c_) are selected to be higher than the values at the frequency of the input pulses, allowing these signals to pass through the inductor (*L*_1_), transistor (*M*_B1_), diode (*D*_B1_ and *D*_B2_), and capacitor (*C*_B1_) to ground:

(1) $$f_{c}=\frac{1}{2\pi }\sqrt{\frac{L_{1}-\left(C_{D1}+C_{B1}\right){\left(C_{D2}+\frac{1}{R_{MR1}}\right)}^{2}}{(C_{D1}+C_{B1})(R_{RB1}+\left(C_{D2}+\frac{1}{R_{MR1}}\right))L_{1}}}$$

where *R_MR_*_1_ is the parasitic resistance of the HV MOSFET, and *C_D_*_1_ and *C_D_*_2_ are the parasitic capacitances of the diodes (*D*_B1_ and *D*_B2_).

The drain-source parasitic resistance of the HV MOSFET in the on-state can be calculated as

(2) $$R_{MR1}=\frac{\left(V_{GSN}-V_{THN}\right)}{g_{fs}}$$

where *g_fs_* is the forward transconductance, *V_GSN_* is the gate–source voltage, and *V_THN_* is the gate–source threshold voltage.

Thus, the bias-voltage stabilizer designed is expected to block unwanted input pulses in the frequency range defined in (1). As shown in Figure 2a, we selected an HV transistor (*M*_B1_) with relatively low parasitic capacitances (*C*_gd_, *C*_gs_, and *C*_ds_ = 7 pF, 28 pF, and 18 pF, respectively) because the DC voltage from the resistor divider turns on the transistor (*M*_B1_), and bipolar or unipolar pulses from the input port can subsequently pass through the diodes (*D*_B2_ and *D*_B1_) and the capacitor (*C*_B1_) to ground.

## 3. Results and Discussion

**Figure 3:**
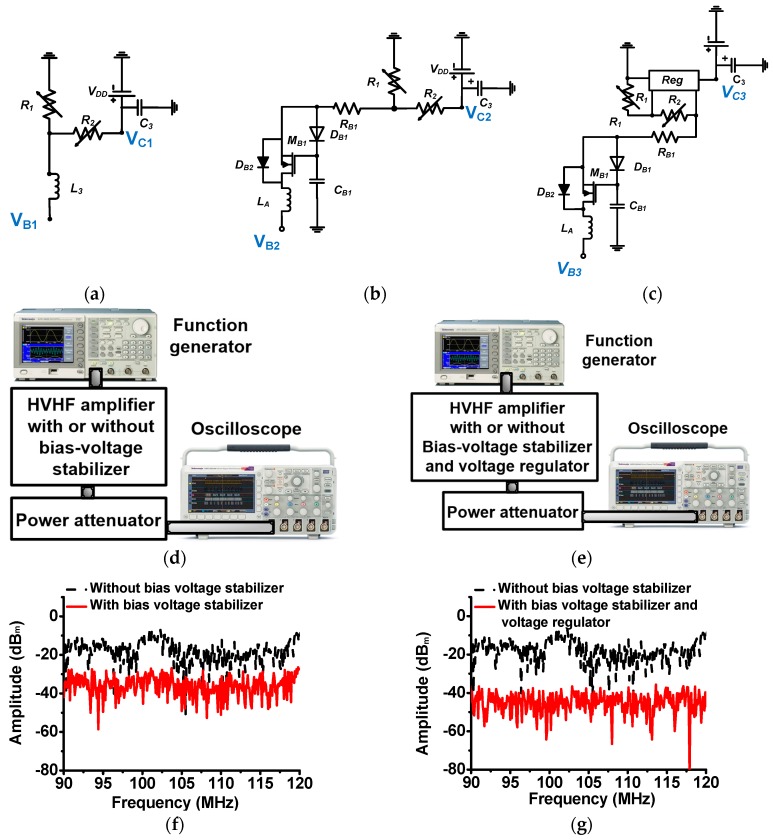
Illustration of the flow of unwanted input pulses through the bias-voltage stabilizer, and measured radio frequency (RF) spectrum data of leakage voltage. Schematics of the (**a**) resistor-divider circuit; (**b**) the resistor divider with a bias-voltage stabilizer; and (**c**) the resistor divider with a bias-voltage stabilizer and voltage regulator; Setup for measuring the leakage voltage of (**d**) the HVHF amplifier with a bias-voltage stabilizer; and (**e**) the HVHF amplifier with a bias-voltage stabilizer and voltage regulator; Measured leakage voltage spectra at the output port (**f**) with and without a bias-voltage stabilizer; and (**g**) with and without a bias-voltage stabilizer and voltage regulator.

Figure 3a–c show the resistor-divider circuit of the HVHF amplifier, the circuit connected to the bias-voltage stabilizer, and the circuit connected to the bias-voltage stabilizer and voltage regulator, respectively. As described in Section 2, the large inductor (*L*_3_) can attenuate unwanted input pulses from the input port. However, these attenuated pulses can still pass through the resistor (*R*_2_) to the power supply, thus affecting the performance of the DC supply. Without a bias-voltage stabilizer, the leakage current can also pass through the resistor divider connected to the DC power supply, resulting in the amplifier operating in over-current and over-heat conditions. To estimate the effect of the bias-voltage stabilizer, we measured and compared the magnitude of the leakage voltages from the input pulses in the different circuit configurations. Figure 3d,e show the experimental setup used to measure the voltage leakage effects. The 40-dB and 50-Ω power attenuator (BW-N40W50+, Mini-Circuits, Brooklyn, NY, USA) was used to protect the oscilloscope (MSOX4154A, Keysight Technology, Santa Clara, CA, USA). As shown in Figure 3f,g, the magnitudes of the leakage voltage obtained using the bias-voltage stabilizer (−26.39 dBm), and using the bias-voltage stabilizer and voltage regulator (−38.45 dBm) were substantially lower than the value obtained without a bias-voltage stabilizer (−7.48 dBm) when bipolar pulses were applied from the input ports (*V*_B1_, *V*_B2_, and *V*_B3_). Hence, leakage voltage from the bias-voltage stabilizer circuit apparently minimizes unwanted input pulses. Thus, with a bias-voltage stabilizer circuit, it is possible to stabilize the DC bias generated from the resistor divider across a wide input power for the transistor in the HVHF amplifier.

**Figure 4:**
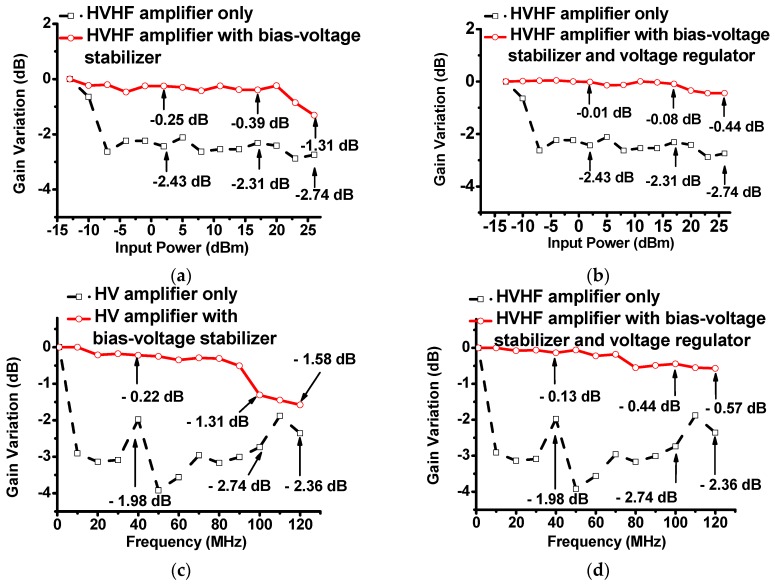
(**a**) Plots of the measured voltage gain deviation vs. input power of the HVHF amplifier alone, and the amplifier with a bias-voltage stabilizer; (**b**) Plots of the measured voltage gain deviation vs. input power of the HVHF amplifier alone, and the amplifier with a bias-voltage stabilizer and voltage regulator; (**c**) Plots of the measured gain deviation vs. frequency of the HVHF amplifier alone, and the HVHF amplifier with a bias-voltage stabilizer; (**d**) Plots of the measured voltage gain deviation vs. frequency of the HVHF amplifier alone, and the HVHF amplifier with the bias-voltage stabilizer and voltage regulator with an input power of 26 dBm.

Figure 4a,b show the plots of the gain deviation of the HVHF amplifier with respect to the input powers, with and without the bias-voltage stabilizer, and with and without the bias-voltage stabilizer and voltage regulator, respectively. In these experiments, five cycles of 100-MHz pulses were applied to the circuits. The bias-voltage stabilizer was excited with DC voltage from a power supply (E3631A, Agilent Technologies, Santa Clara, CA, USA). The AC input of the HVHF amplifier was supplied from a function generator (AFG3252, Tektronix Inc., Beaverton, OR, USA). The output power of the HVHF amplifier was measured using an oscilloscope (MSOX4154A, Keysight Technology, Santa Rosa, CA, USA). Figure 4a illustrates that the gain deviations of the HVHF amplifier, the amplifier with the bias-voltage stabilizer, and amplifier with the bias-voltage stabilizer and voltage regulator were measured. With the bias-voltage stabilizer, the gain deviations of the HVHF amplifier (−0.25 dB, −0.39 dB, and −1.31 dB, respectively) were lower than those of the HVHF amplifier alone (−2.43 dB, −2.31 dB, and −2.74 dB, respectively), at input powers of 2 dBm, 17 dBm, and 26 dBm, as can be seen through a comparison of Figure 4a,b. At the previously stated input powers, the gain deviations of the HVHF amplifier with a bias-voltage stabilizer and voltage regulator (−0.01 dB, −0.08 dB, and −0.44 dB, respectively) were also lower than those of the HVHF amplifier without additional circuitry. These results indicate that the stabilizer can reduce variations in the bias voltage of the HVHF amplifier in the −10 dBm to 26 dBm input range. In addition, the combination of a bias-voltage stabilizer and a voltage regulator in a HVHF amplifier can further reduce the voltage gain deviations at high power levels (up to 26 dBm).

Figure 4c,d show the plot of the gain deviation of the HVHF amplifier with respect to frequency with and without the bias-voltage stabilizer, and with and without the bias-voltage stabilizer and voltage regulator, respectively. In these experiments, five cycles of input pulses with a power of 26 dBm were applied to the circuits. Figure 4c,d illustrate that with 10 MHz input pulses, the gain deviations of the HVHF amplifier only, the amplifier with the bias-voltage stabilizer, and the amplifier with the bias-voltage stabilizer and voltage regulator were measured. With an input power of 26 dBm and a frequency of 120 MHz, the gain deviations of the HVHF amplifier alone and the amplifier with the bias-voltage stabilizer were measured to be −2.36 dB and −1.58 dB, respectively, as depicted in Figure 4c. The gain deviation of the HVHF amplifier with the bias-voltage stabilizer and voltage regulator was measured as −0.57 dB, with the input power defined as before, as depicted in Figure 4d. The characteristics of the HVHF amplifier without voltage stabilization or regulation, which mirror the plot in Figure 4c, are also included for comparative purposes. These results indicate that the proposed stabilizer can reduce variations in the bias voltage of the HVHF amplifier in the frequency range between 1 MHz and 120 MHz. In addition, the combination of the bias-voltage stabilizer with the voltage regulator can further reduce voltage gain deviations at 120 MHz, compared to the sole use of the bias-voltage stabilizer, with an HVHF amplifier.

**Figure 5:**
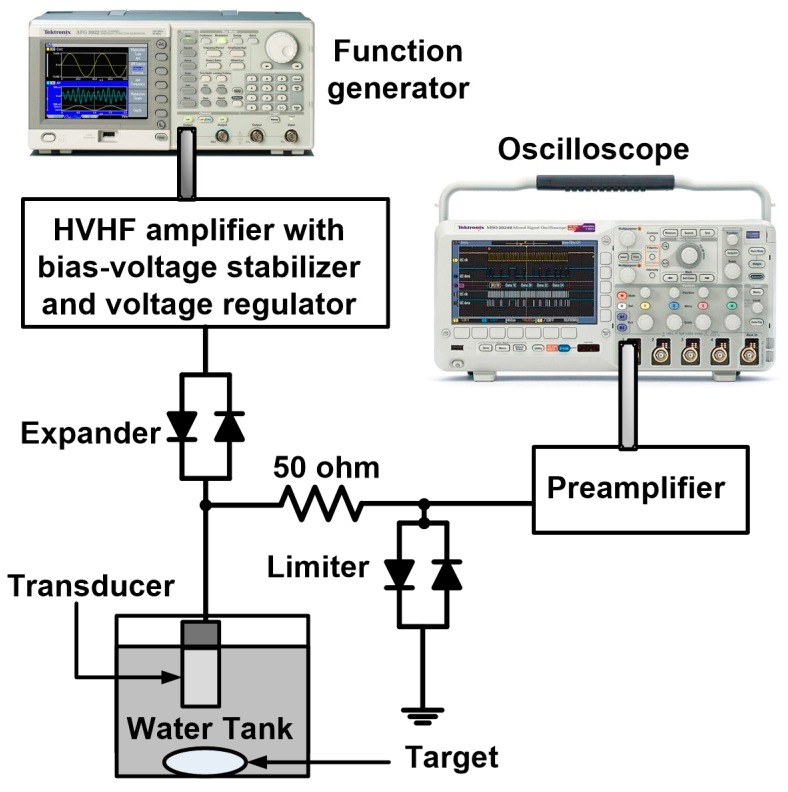
Experimental setup of a very-high-frequency (VHF) pulse-echo measurement system using the HVHF amplifier with and without a bias-voltage stabilizer and voltage regulator.

Table 1 summarizes the measured performances of the HVHF amplifier, HVHF amplifier with bias-voltage stabilizer, and HVHF amplifier with bias-voltage stabilizer and voltage regulator. To verify the feasibility of deploying the bias-voltage stabilizer circuit in VHF ultrasound transducer applications, we implemented a typical pulse-echo measurement system, as shown in Figure 5. This system is typically used to evaluate the operation of ultrasound transducers or electronic devices developed for ultrasound applications [26,27]. Five cycles of sinusoidal bipolar pulses, generated by a function generator (AFG3252), were transmitted through the proposed HVHF amplifier, the amplifier with the bias-voltage stabilizer, and the amplifier with the bias-voltage stabilizer and a voltage regulator, to trigger a focused immersion transducer through a diode-based expander (a cross-coupled diode, P1N4148, Diodes Incorporated, Plano, TX, USA). The ultrasound transducer detected the echo signals from the target. The received echo signals were then transmitted through a diode-based limiter (a 50-Ω shunt resistor with a cross-coupled diode) with a preamplifier (AU-1114, MITEQ Inc., Hauppauge, NY, USA). Waveforms of the echo signals were displayed on a 1-GHz 5-GS/s sampling oscilloscope (MSOX4154A, Keysight Technology). Subsequently, the spectral data from the echo signals was processed using a MATLAB program (MathWorks Inc., Natick, MA, USA) on a personal computer.

**Figure 6:**
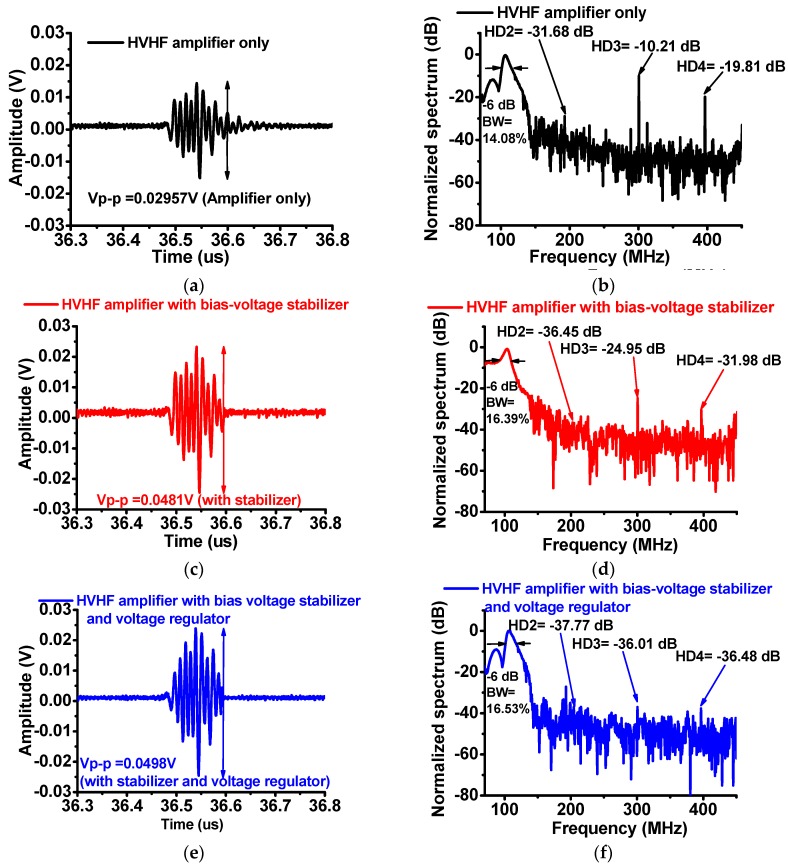
Amplitudes of echo signals and normalized spectra from the 100-MHz transducer. (**a**) Amplitude and (**b**) spectra of the echo signal of the HVHF amplifier alone; (**c**) Amplitude and (**d**) spectra of the echo signal of the amplifier with the bias-voltage stabilizer; (**e**) Amplitude and (**f**) spectra of the echo signal of the amplifier with a voltage regulator and the bias-voltage stabilizer.

Figure 6 shows a comparison of the waveforms of the echo signals and the spectra generated by the immersion transducer triggered using the HVHF amplifier alone, the amplifier with the bias-voltage stabilizer, and the HVHF amplifier with the bias-voltage stabilizer and a regulator. Figure 6a,c,e show that the measured amplitudes of the echo signals from the HVHF amplifier with the bias-voltage stabilizer (0.0481 V_p-p_) and the amplifier with the bias-voltage stabilizer and regulator (0.0498 V_p-p_) are higher than that of the HVHF amplifier alone (0.0296 V_p-p_). Figure 6b,d,f show that the second, third, and fourth harmonic-distortion components (HD2 = −36.45 dB, HD3 = −24.95 dB, and HD4 = −31.98 dB) of the signals generated by the HVHF amplifier with the bias-voltage stabilizer, and the second, third, and fourth harmonic-distortion components (HD2 = −37.77 dB, HD3 = −36.01 dB, and HD4 = −36.48 dB) of the signals generated by the amplifier with the bias-voltage stabilizer and voltage regulator were lower than those of the signals generated by the HVHF amplifier alone (HD2 = −31.68 dB, HD3 = −10.21 dB, and HD4 = −19.81 dB). The −6-dB bandwidths (BWs) of the echo signals of the HVHF amplifier alone, the HVHF amplifier with the bias-voltage stabilizer, and the HVHF amplifier with a voltage-regulator and bias-voltage stabilizer are 14.08%, 16.39%, and 16.53%, respectively. The total harmonic distortions of the echo signals calculated for situations when the transducer is triggered by the HVHF amplifier alone, the amplifier with the bias-voltage stabilizer, and with the bias-voltage stabilizer and a voltage regulator, were −19.46 dB, −47.83 dB, and −63.84 dB, respectively. These results thus demonstrate that HVHF amplifiers with a bias-voltage stabilizer, or with a bias-voltage stabilizer and a voltage regulator, may be useful in improving the amplitudes of echo signals and reducing the harmonic distortions of the echo signals from the VHF ultrasound transducers used in pulse-echo measurement systems.

## 4. Conclusions

VHF ultrasound transducers have recently been highlighted for use in various ultrasound applications, such as acoustic microscopy and micro-beam stimulation. In the pulse-echo measurement systems, the magnitudes of echo signals from VHF ultrasound transducers are required to be lower than those from LF ultrasound transducers, owing to factors related to the inherent device architecture, such as the size of the transducers, and a limited range of excitation voltages. Hence, to obtain stable echo-signal amplitudes from VHF transducers, the performance of the HVHF amplifier is crucial. Therefore, we proposed the use of a bias-voltage circuit to stabilize the DC bias voltages of the HVHF amplifiers and subsequently reduce the magnitude of the harmonic-distortion components of the echo signals in the pulse-echo measurement systems.

Using the proposed bias-voltage stabilizer circuit, stable input bias voltages were generated from the wider input ranges of the HVHF amplifier, thus yielding reduced harmonic distortions in the echo signals. To confirm the feasibility of the bias-voltage stabilizer for VHF ultrasound transducers, the quality of an echo signal from a VHF immersion ultrasound transducer was obtained and compared using a pulse-echo measurement system. The amplitudes of the echo signals obtained with a bias-voltage stabilizer (0.0481 V_p-p_) and with the bias-voltage stabilizer and voltage regulator (0.0498 V_p-p_) were higher than the amplitude obtained without a bias-voltage stabilizer (0.0296 V_p-p_). Using the bias-voltage stabilizer, and the bias-voltage stabilizer and a regulator, the second, third, and fourth harmonic-distortion performances improved in comparison to using the HVHF amplifier alone. Thus, the bias-voltage stabilizer scheme presented is a potential method for stabilizing the performance of HVHF amplifiers used in VHF pulse-echo measurement systems, thus generating low harmonic distortions for the echo signals.

## Figures and Tables

**Table 1:** Summarized performances of the (**a**) HVHF amplifier alone; (**b**) the amplifier with the bias-voltage stabilizer; and (**c**) the amplifier with the bias-voltage stabilizer and a voltage regulator, along with test conditions.

	(a)	(b)	(c)
Average noise level (dBm)	−7.48	−26.39	−38.45
Gain deviation (dBm) with a 17-dBm input at 100 MHz	−2.31	−0.39	−0.08
Gain deviation (dBm) with a 26-dBm input at 100 MHz	−2.74	−1.31	−0.44
